# Supplementary material for: Resolving the full spectrum of human genome variation using Linked-Reads
Source: Genome Res. 2019 Apr;29(4):635–45. doi: 10.1101/gr.234443.118 (PMC6442396; doi:10.1101/gr.234443.118)
Supplement: Supplemental Material [file supp_gr.234443.118_Supplemental_longranger-2.2.zip › longranger-2.2.tar.bz2!/lr2.2-public/lib/stan/cmdstan/stan/doc/api/html/structstan_1_1gm_1_1variable__map.html]

Stan: stan::gm::variable\_map Struct Reference


|  |  |
| --- | --- |
|  | Stan  2.5.0  probability, sampling & optimization |


- Main Page
- Related Pages
- Namespaces
- Classes
- Files

- Class List
- Class Index
- Class Hierarchy
- Class Members

All Classes Namespaces Files Functions Variables Typedefs Enumerations Enumerator Friends Macros Pages

- stan
- gm
- variable\_map

Public Types |
Public Member Functions |
Public Attributes |
List of all members

stan::gm::variable\_map Struct Reference

`#include <ast.hpp>`

|  |  |
| --- | --- |
| Public Types | |
| typedef std::pair  < base\_var\_decl, var\_origin > | range\_t |
|  | |

|  |  |
| --- | --- |
| Public Member Functions | |
| bool | exists (const std::string &name) const |
|  | |
| base\_var\_decl | get (const std::string &name) const |
|  | |
| base\_expr\_type | get\_base\_type (const std::string &name) const |
|  | |
| size\_t | get\_num\_dims (const std::string &name) const |
|  | |
| var\_origin | get\_origin (const std::string &name) const |
|  | |
| void | add (const std::string &name, const base\_var\_decl &base\_decl, const var\_origin &vo) |
|  | |
| void | remove (const std::string &name) |
|  | |

|  |  |
| --- | --- |
| Public Attributes | |
| std::map< std::string, range\_t > | map\_ |
|  | |

## Detailed Description

Definition at line 439 of file ast.hpp.

## Member Typedef Documentation

typedef std::pair<base\_var\_decl,var\_origin> stan::gm::variable\_map::range\_t

Definition at line 440 of file ast.hpp.

## Member Function Documentation

|  |  |  |  |
| --- | --- | --- | --- |
| void stan::gm::variable\_map::add | ( | const std::string & | *name*, |
|  |  | const base\_var\_decl & | *base\_decl*, |
|  |  | const var\_origin & | *vo* |
|  | ) |  |  |

Definition at line 1007 of file ast\_def.cpp.

|  |  |  |  |  |  |
| --- | --- | --- | --- | --- | --- |
| bool stan::gm::variable\_map::exists | ( | const std::string & | *name* | ) | const |

Definition at line 988 of file ast\_def.cpp.

|  |  |  |  |  |  |
| --- | --- | --- | --- | --- | --- |
| base\_var\_decl stan::gm::variable\_map::get | ( | const std::string & | *name* | ) | const |

Definition at line 991 of file ast\_def.cpp.

|  |  |  |  |  |  |
| --- | --- | --- | --- | --- | --- |
| base\_expr\_type stan::gm::variable\_map::get\_base\_type | ( | const std::string & | *name* | ) | const |

Definition at line 996 of file ast\_def.cpp.

|  |  |  |  |  |  |
| --- | --- | --- | --- | --- | --- |
| size\_t stan::gm::variable\_map::get\_num\_dims | ( | const std::string & | *name* | ) | const |

Definition at line 999 of file ast\_def.cpp.

|  |  |  |  |  |  |
| --- | --- | --- | --- | --- | --- |
| var\_origin stan::gm::variable\_map::get\_origin | ( | const std::string & | *name* | ) | const |

Definition at line 1002 of file ast\_def.cpp.

|  |  |  |  |  |  |
| --- | --- | --- | --- | --- | --- |
| void stan::gm::variable\_map::remove | ( | const std::string & | *name* | ) |  |

Definition at line 1012 of file ast\_def.cpp.

## Member Data Documentation

std::map<std::string, range\_t> stan::gm::variable\_map::map\_

Definition at line 452 of file ast.hpp.

---

The documentation for this struct was generated from the following files:

- src/stan/gm/ast.hpp
- src/stan/gm/ast\_def.cpp


---

[ Stan Home Page ]

*© 2011–2014,
Stan Development Team.*
